# Evaluation of psychological changes using facial emotion analysis in postoperative rehabilitation treatment for patients with upper gastrointestinal cancer: A prospective study

**DOI:** 10.1371/journal.pone.0340914

**Published:** 2026-01-20

**Authors:** Naoto Seriu, Shogo Sasaki, Yukako Ishida, Yasuyo Kobayashi, Tetsuro Kitamura, Yuya Mawarikado, Yosuke Uchihashi, Yusuke Inagaki, Masayuki Sho, Akira Kido

**Affiliations:** 1 Department of Rehabilitation Medicine, Nara Medical University, Kashihara, Nara, Japan; 2 Medicinal Biology of Thrombosis and Haemostasis, Nara Medical University, Kashihara, Nara, Japan; 3 Department of Surgery, Nara Medical University, Kashihara, Nara, Japan; Aichi Prefectural Mikawa Aoitori Medical and Rehabilitation Center for Developmental Disabilities, JAPAN

## Abstract

Rehabilitation after radical gastrectomy or esophagectomy for upper gastrointestinal cancer can improve physical function and quality of life; however, objective day-to-day measures of psychological change are lacking. We aimed to test whether facial emotion analysis can quantitatively evaluate patients’ emotional responses before and after each rehabilitation session and whether these changes relate to conventional subjective/physiological stress markers and discharge physical outcomes. We conducted a single-center prospective observational study of 32 patients who underwent radical gastrectomy or esophagectomy between August 2024 and February 2025. Immediately before and after each rehabilitation session, 30 s iPad video interviews (median, six per patient) were recorded and analyzed using MAL Face Emotion software to obtain normalized scores (0%–100%) for Neutral, Happy, Sad, Angry, and Surprised emotions. Subjective stress (0–100 mm visual analog scale) and salivary α-amylase activity were collected concurrently; discharge physical function was assessed using the 6 min walk distance and Five Times Sit-to-Stand tests. Pre- and post-session values were compared using Wilcoxon signed-rank tests, and associations were examined with age-adjusted regression and Spearman correlation. Thirty-one patients completed the study without adverse events. After rehabilitation, the Happy score increased (median +3.5%, p = 0.013) and stress decreased (−1.5 mm, p = 0.025), whereas salivary α-amylase and other emotions were unchanged. Changes in the Happy score (p = 0.21) and stress (p = 0.19) did not predict discharge physical function, whereas changes in the Sad score correlated moderately with changes in salivary α-amylase (ρ = 0.45). The findings of this single-center study provide preliminary evidence supporting the feasibility of facial emotion analysis as a non-invasive, quantitative tool for real-time psychological monitoring during postoperative rehabilitation. Furthermore, our results demonstrate its potential to support a more personalized delivery of cancer rehabilitation.

## Introduction

Surgical therapy is an indispensable treatment for patients with gastric or esophageal cancer [1–3]. Because physical function and quality of life (QOL) decline from the early postoperative period, commencing rehabilitation treatment immediately after surgery is crucial [4]. In recent years, the reduction in the invasiveness of surgical techniques has been met with the introduction of Enhanced Recovery After Surgery (ERAS) programs that include walking training from the day following surgery [5–8]. These programs have led to a reduction in respiratory and severe postoperative complications [9]; moreover, they have been shown to improve both cardiopulmonary function and QOL [10]. Thus, postoperative rehabilitation benefits not only physical function but also psychological function. However, most previous studies have assessed QOL using subjective self-report methods [10–12]. QOL assessments are routinely conducted at regular intervals (e.g., weekly or monthly). In cancer rehabilitation, instruments such as the European Organization for Research and Treatment of Cancer Quality of Life Questionnaire Core 30 [13] and Functional Assessment of Cancer Therapy [14] are widely used. In validation studies, these questionnaires specify a recall period of the past week only. Thus, they are not considered suitable for capturing day-to-day in-hospital changes. Moreover, to the best of our knowledge, no studies in postoperative rehabilitation for gastrointestinal cancer have objectively and longitudinally tracked daily psychological changes during inpatient stays.

In recent years, rapid advances in deep learning have expanded what can be quantified from complex biomedical and sensor data, which has enabled analyses that were previously considered impractical [15,16]. Building on these advances, facial emotion analysis has been explored in clinical contexts. For example, facial emotion analysis, as a clinical application, revealed that patients with coronary artery disease show an increase in negative emotions and a decrease in positive emotions during cardiopulmonary exercise testing [17]. Another study evaluated the facial expressions of 10 patients with traumatic brain injury while performing speech tasks and found that the proportions of both engagement time (defined by multiple facial action units) and smiling time were significantly higher in patients with traumatic injury than in control participants [18]. The more frequent use of facial expressions by the traumatic brain injury patients may have compensated for communication impairments, such as reduced language output and memory function. Additionally, facial emotion analysis of 210 lung cancer patients during preoperative health education showed that the content, tone, and vocabulary of the educational sessions influenced patients’ emotional responses [19]. Considering these findings, we reasoned that automated facial emotion analysis, an objective, quantitative, and non-invasive approach, could help characterize patients’ emotional states during rehabilitation. Because it allows near real-time estimation of emotional expressions, the integration of facial emotion analysis into rehabilitation may help guide therapists to effectively adjust intensity and task selection.

The purpose of this study was to use facial emotion analysis during postoperative inpatient rehabilitation to capture day-to-day psychological changes during hospitalization. Specifically, we sought to describe these changes and examine their associations with physical function as well as conventional subjective and physiological assessments. We hypothesized that facial emotion analysis would be associated with patient-reported stress and functional status, and could serve as a complementary objective indicator to support effective rehabilitation. The novelty of this study lies in introducing artificial intelligence (AI)-based facial emotion analysis into postoperative cancer rehabilitation.

## Materials and methods

### Ethics statement

This study complied with the Declaration of Helsinki and was approved by the Ethics Committee of Nara Medical University (approval number 3751). All participants received both oral and written explanations of the study and provided written informed consent before enrollment.

### Study design and participants

In this single‑center, prospective, observational study, we enrolled patients admitted to Nara Medical University Hospital between August 2024 and February 2025 who met the following inclusion criteria: (1) a diagnosis of gastric or esophageal cancer, including tumors of the esophagogastric junction; (2) receipt of radical gastrectomy or esophagectomy; (3) were aged 18 years or over at the time of surgery; and (4) ability to ambulate in the perioperative period. Exclusion criteria were: (1) a history of significant facial trauma or facial nerve palsy; (2) severe aphasia or dysarthria that hindered communication; (3) severe psychiatric illness; or (4) an infection requiring isolation during hospitalization. Basic patient information was obtained from electronic medical records: sex, age, tumor site, tumor stage, presence or absence of neoadjuvant therapy, surgical approach, operative time, blood loss, preoperative parameters (forced expiratory volume in 1 s/forced vital capacity ratio [FEV₁/FVC], hemoglobin level, C reactive protein level, and serum albumin level), severity of postoperative complications according to the Clavien–Dindo classification, and length of hospital stay.

### Facial emotion analysis

Facial emotion analysis of recorded video was conducted using the MAL Face Emotion software (Vitalify Asia Co., Ltd., Ho Chi Minh City, Vietnam). The software’s artificial intelligence model identifies facial expressions in each frame and provides a real‑time numerical score on a 0–100 scale for five emotion categories: Neutral, Happy, Sad, Angry, and Surprised. These scores are then normalized to percentages that sum to 100%. Facial expressions can be detected at 15–20 frames/s. The underlying model employs a MobileNets architecture with depth‑separable convolutions for face recognition [20] and is trained primarily on the AffectNet database [21]. We recorded the videos using an iPad (Apple Inc., Cupertino, CA, USA) and analyzed the resulting footage. We performed facial emotion analysis during the 30 s interviews conducted before and after each rehabilitation session. Before each session, patients were asked about their physical condition, sleep status, and food intake for that day, and exercise intensity was adjusted accordingly. After the session, physiotherapists inquired about fatigue, physical condition relative to the training load, and impressions of their task performance. These questions were part of routine clinical practice and were thus not expected to influence emotional status.

### Conventional assessments

Subjective psychological stress was measured using a visual analog scale (VAS) ranging from 0 (no stress) to 100 mm (maximum stress). Salivary α‑amylase activity (sAA) was assessed using a salivary amylase monitor (Nipro Co., Osaka, Japan) as a physiological stress marker. In previous studies, sAA has been validated as a noninvasive indicator of mental stress [22] and applied to cancer populations [23–25]. VAS scores and sAA levels were measured before and after each rehabilitation session, in parallel with facial emotion analysis.

### Physical function

Physical function was evaluated using the 6 min walk distance (6MWD) and Five Times Sit-to-Stand tests (FTSST). The 6MWD test follows the American Thoracic Society guidelines [26], whereby participants are instructed to walk back and forth at the maximum safe speed along a flat, straight course. The FTSST measures the time required to rise five times from a 40 cm-high chair with arms folded and is a low‑cost and highly reliable test of lower‑limb strength and dynamic balance for both healthy individuals and patients [27].

### Perioperative rehabilitation protocol

Patients were admitted 1–3 days before surgery and received explanations of the procedure and postoperative care from the surgical team and nursing staff. On the first postoperative day, all patients were referred to the Department of Rehabilitation Medicine and examined by a physiatrist. Thereafter, a physiotherapist initiated early mobilization training, which included sitting, standing, and walking exercises. Each rehabilitation session lasted 20–40 min and was conducted five times per week. The physiotherapist tailored a rehabilitation program that combined lower‑limb strength training and aerobic exercise according to each patient’s condition and postoperative recovery.

### Statistical analysis

All data are presented as medians and interquartile ranges (IQRs). Changes in each emotion category, VAS score, and sAA level following rehabilitation sessions were assessed using the Wilcoxon signed‑rank test. For the emotion-related factors that showed a significant change following rehabilitation, we calculated the rate of change from before to after the treatment and used multiple regression analysis (adjusting for age only) to assess how the change rate influences performance on the 6MWD test and the FTSST at discharge, taking into account the limited sample size. To determine the relationship between facial emotion analysis and conventional measures (i.e., VAS scores and sAA levels), we calculated Spearman’s rank correlation coefficients. All statistical analyses were performed using JMP Pro 17.2.0 software (SAS Institute Inc., Cary, NC, USA), and significance was defined as p < 0.05. All analyses were exploratory, and no adjustment was made for multiple comparisons.

## Results

The median age of the 32 enrolled patients was 75.5 years (IQR 71–79.3 years), and 22 (69%) were men (Table 1). All patients underwent curative‑intent surgery, although one patient was found to have stage IV disease on final pathology. All procedures were performed using minimally invasive techniques, which included robot‑assisted or laparoscopic approaches. Preoperatively, 10 patients had a FEV₁/FVC ratio below 70%, which indicated obstructive ventilatory impairment. One patient experienced a postoperative stroke (Clavien–Dindo classification ≥ 3) with resultant hemiplegia and dysarthria on postoperative day 1 and was thus excluded, which resulted in 31 patients for the facial emotion analysis. The remaining 31 patients completed a median of nine rehabilitation sessions (IQR 7–11) without adverse events.

**Table 1:** Baseline characteristics of the 32 enrolled patients.

	All participants (n = 32)
Sex, n (%)	
Male	22 (69)
Female	10 (31)
Age, years	75.5 (71–79.3)
Tumor location	
Esophagus	9 (28)
Esophagogastric junction	2 (6)
Stomach	21 (66)
Tumor stage (TNM)	
I	13 (41)
II	9 (28)
III	9 (28)
IV	1 (3)
Neoadjuvant treatment, n (%)	
Yes	12 (37.5)
No	20 (62.5)
Type of surgery	
RAMIE	9 (28.1)
LPG	1 (3.1)
LDG	6 (18.8)
LTG	1 (3.1)
RPG	6 (18.8)
RDG	4 (12.5)
RTG	4 (12.5)
PD	1 (3.1)
Operation time, min	414 (310–519)
Blood loss, ml	35 (10–90)
Preoperative parameters	
FEV₁/FVC ratio, %	72.36 (67.90–77.58)
Hemoglobin, g/dL	11.35 (10.4–12.3)
CRP, mg/dL	0.11 (0.03–0.40)
Albumin, g/dL	3.8 (3.5–4.0)
Significant postoperative complications	
Clavien–Dindo Classification≦2	31 (97)
Clavien–Dindo Classification≧3	1 (3)
6MWD test at discharge, m (n = 27)	417 (365–459)
FTSST at discharge, s (n = 28)	10.22 (8.04–12.62)
Length of stay, days	12 (11–19)

Data are presented as numbers (%) or medians (IQRs). Tumor stage refers to the pathological TNM classification.

Abbreviations: RAMIE, robot-assisted minimally invasive esophagectomy; LPG, laparoscopic proximal gastrectomy; LDG, laparoscopic distal gastrectomy; LTG, laparoscopic total gastrectomy; RPG, robotic proximal gastrectomy; RDG, robotic distal gastrectomy; RTG, robotic total gastrectomy; PD, pancreatoduodenectomy; CRP, C-reactive protein

**Fig 1:**
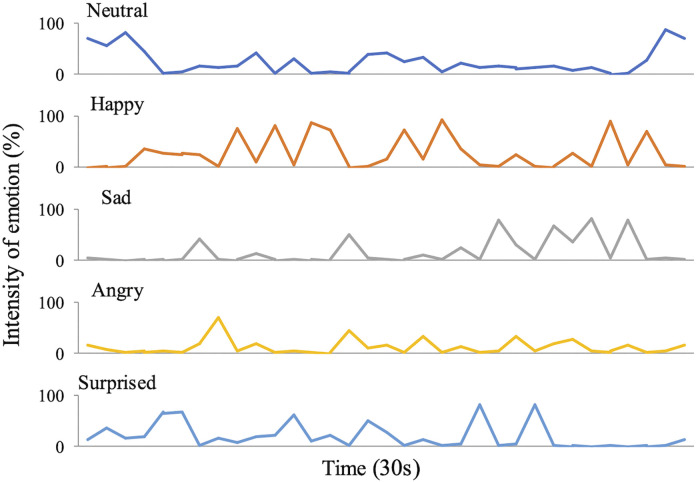
Representative raw emotion time series for one participant. Neutral, Happy, Sad, Angry, and Surprised intensities (0%–100%) were sampled at 15–20 frames/s during the 30 s before and after each rehabilitation session.

**Fig 2:**
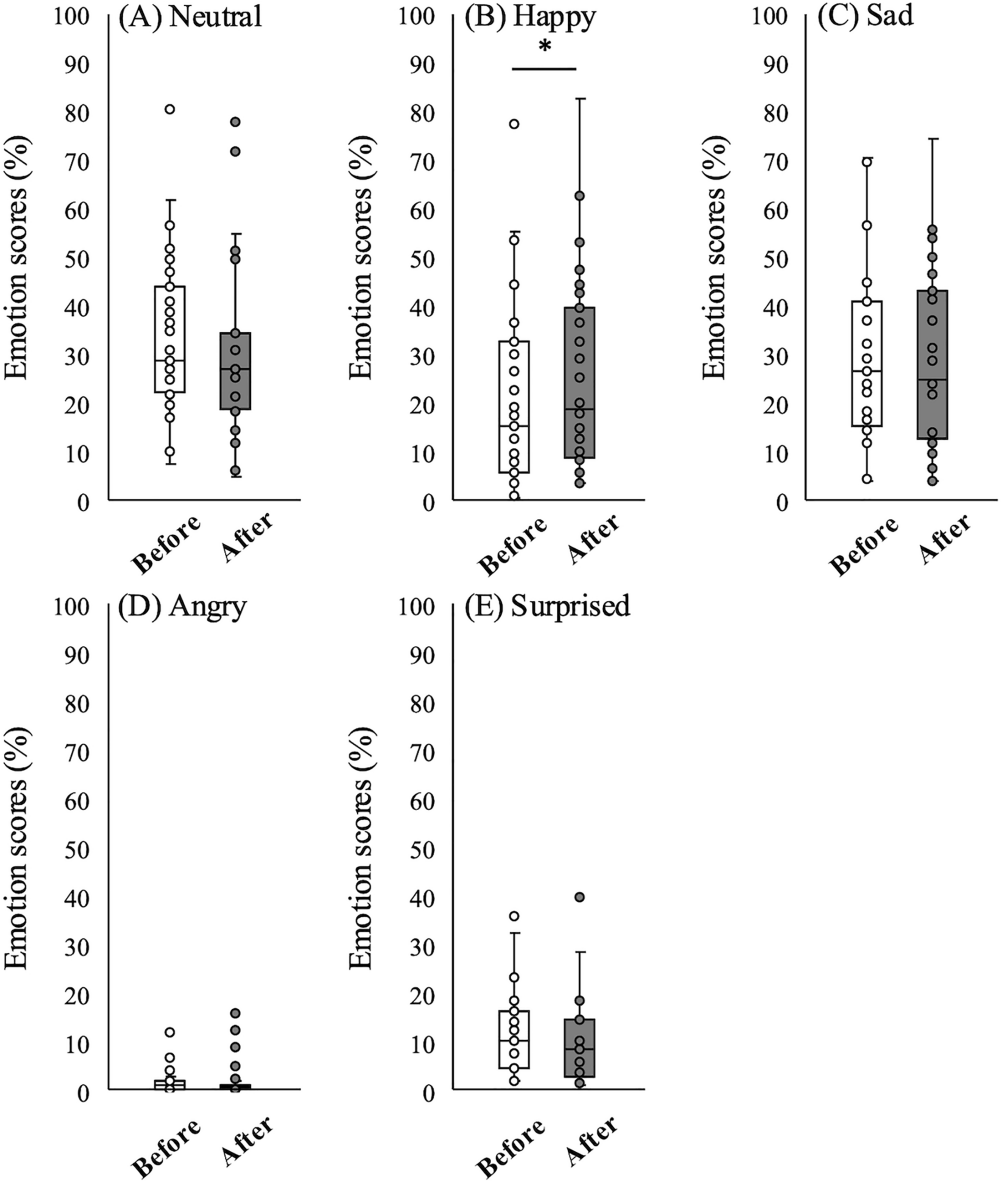
Distribution of emotion scores before and after rehabilitation sessions. Box plots of emotion scores before (white) and after (gray) rehabilitation sessions (n = 31): **(A)** Neutral, **(B)** Happy, **(C)** Sad, **(D)** Angry, and **(E)** Surprised. *p < 0.05.

**Fig 3:**
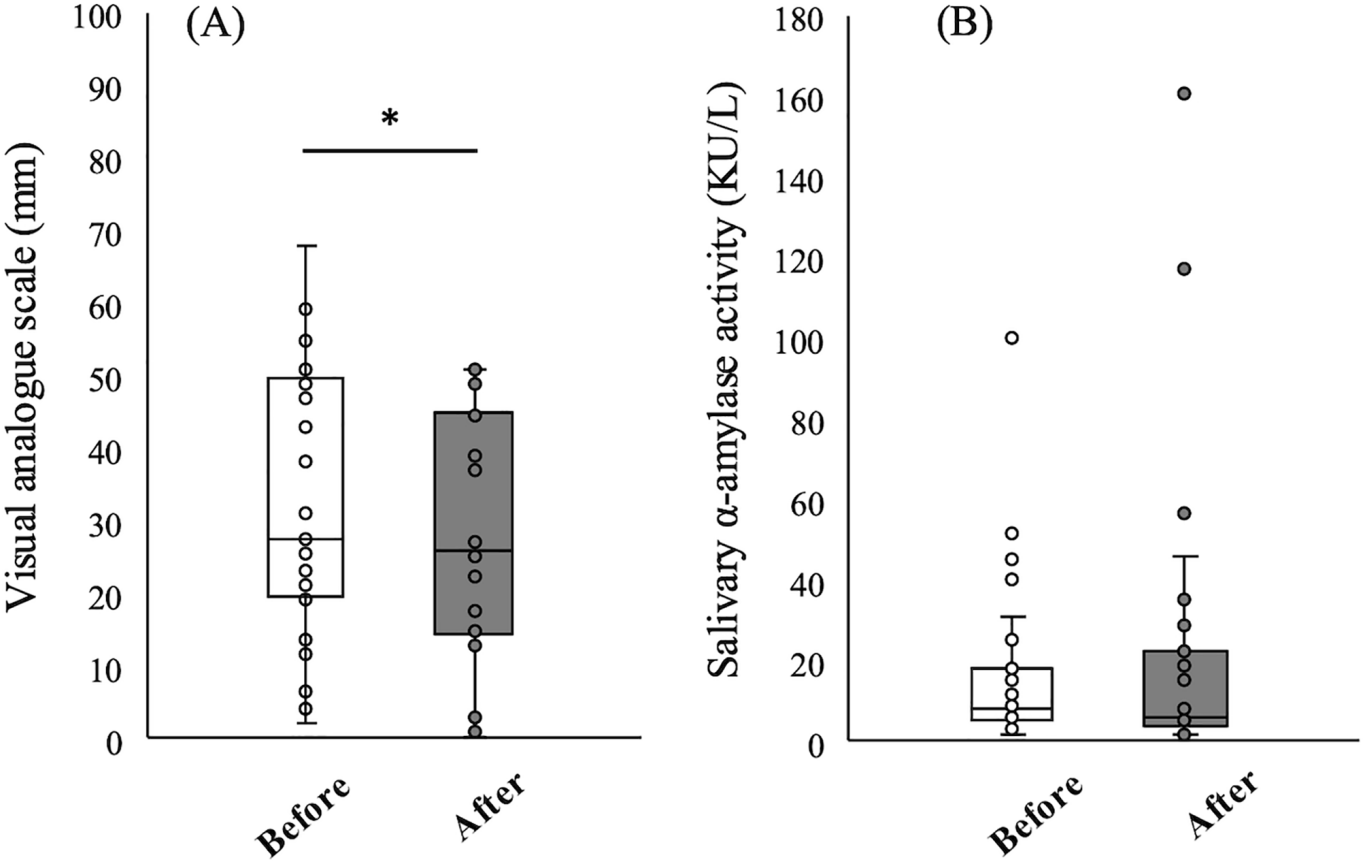
Changes in subjective and physiological stress markers before and after rehabilitation sessions. Box plots of stress markers before (white) and after (gray) rehabilitation sessions (n = 31): **(A)** VAS score and **(B)** sAA. *p < 0.05.

Fig 1 shows representative facial emotion analysis data. The graphs illustrate real‑time changes in each emotion category. Recordings were made six times (IQR 5–8.5) during rehabilitation treatment during the interviews conducted immediately before and after each session. Fig 2 shows box plots of emotion scores before and after rehabilitation sessions. The median pre- and post-rehabilitation Happy scores were 15.02 (IQR 5.68–31.25) and 18.48 (IQR 9.41–37.98), indicating a significant increase (Wilcoxon signed-rank test, p = 0.013; Fig 2B). No other emotion scores changed significantly. The median pre- and post-rehabilitation VAS scores were 27.5 (IQR 20–49) and 26.0 (IQR 15–44.5), a significant decrease (Wilcoxon signed-rank test, p = 0.025; Fig 3A), whereas sAA levels did not change (Fig 3B).

Next, we examined the associations between the emotion scores that showed significant pre–post differences (Happy and VAS) and physical function (6MWD and FTSST). Age-adjusted linear regression provided no evidence that changes in Happy or VAS predicted discharge-day performance. For both 6MWD (n = 27) and FTSST (n = 28), all 95% confidence intervals for the coefficients included zero, and the model tests were non-significant (Table 2). Four patients lacked 6MWD data (refusal because of poor condition, n = 3; COVID-19 infection 1 day before discharge, n = 1), and three patients lacked FTSST data (inability to rise from the specified seat height, n = 2; early discharge, n = 1). Thus, the respective analyses comprised 27 and 28 patients.

**Table 2:** Age‑adjusted associations between changes in the Happy emotion and VAS stress scores and the 6MWD test and FTSST performance at discharge.

	6MWD (n = 27)				FTSST (n = 28)			
	Beta	Standard error	95% confidence interval	P-value	Beta	Standard error	95% confidence interval	P-value
Happy score	0.030	0.023	−0.019 to 0.080	0.212	−0.001	0.0008	−0.003 to 0.001	0.240
VAS score	0.339	0.248	−0.176 to 0.854	0.186	−0.003	0.008	−0.021 to 0.015	0.752
Age	−4.543	2.881	−10.519 to 1.431	0.129	0.157	0.101	−0.053 to 0.367	0.135

**Fig 4:**
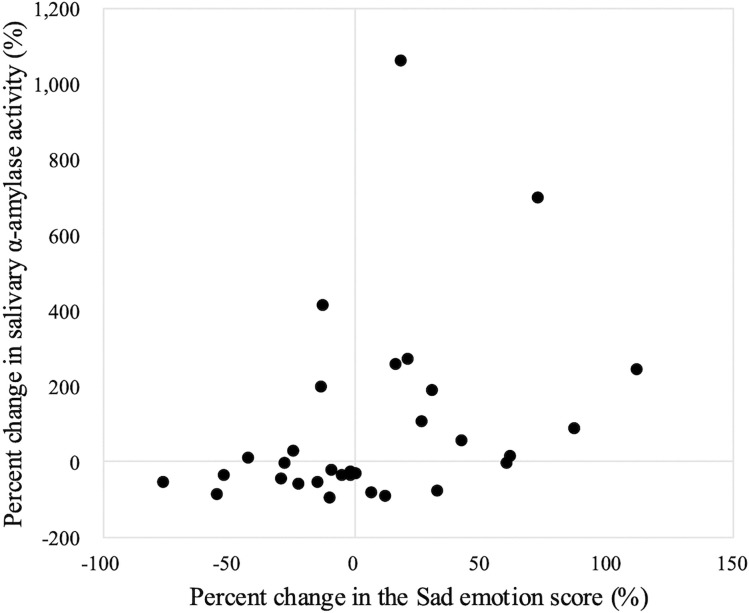
Correlation between percent change in Sad emotion score and sAA level. Each dot represents each patient’s percent change in Sad emotion score as a function of percent change in sAA level (n = 31). Spearman’s rank correlation: ρ = 0.45, p = 0.01.

Next, we investigated the relationship between facial emotion analysis and conventional subjective and physiological assessments. There were no significant associations between the change rate of any of the emotion categories and VAS score change. Change in sAA level showed no significant correlation with Neutral, Happy, Angry, or Surprised scores, but was moderately positively correlated with Sad scores (Spearman rank correlation, ρ = 0.45, p = 0.01; Fig 4).

## Discussion

To the best of our knowledge, this is the first study on cancer rehabilitation to use facial emotion analysis for objective psychological assessment. In gastric and esophageal cancer patients undergoing postoperative rehabilitation, daily evaluations before and after each rehabilitation session revealed significant improvements in the Happy emotion score and VAS stress ratings. Moreover, the change in the Sad emotion score correlated moderately positively with the change in sAA level, a physiological stress biomarker.

There is now considerable research that has applied automated facial emotion analysis to real-world care settings. For neurological rehabilitation, a recent study in patients with mild cognitive impairment and Alzheimer’s disease quantified task-dependent emotion profiles during group sessions and demonstrated the feasibility of repeated, in-session measurement and its utility for tailoring activities [28]. In dementia care facilities, a field experiment using FaceReader captured second-to-minute changes in valence and basic emotions during sound-based interventions and showed partial concordance with contemporaneous self-reports, supporting the ecological validity of automated facial emotion analysis [29]. Beyond rehabilitation, analyses of routine outpatient consultations have modeled patient–clinician affect from clinical videos and revealed systematic emotion dynamics over the course of one visit [30]. Furthermore, perioperative and critical-care studies have indicated that automated facial analysis can classify pain at clinically meaningful levels in adults, including surgical/interventional cohorts and intensive care unit patients, which demonstrates its sensitivity to short-timescale, stress-related affect [31,32]. In oncology specifically, AI-based emotion analysis has been used to evaluate preoperative educational encounters in patients with lung cancer [19]; however, studies linking daily, session-linked emotional changes to postoperative rehabilitation remain scarce. Our work addresses this gap by implementing repeated pre- and post-session measurements during inpatient gastrointestinal cancer rehabilitation and triangulating automated facial emotion analysis using subjective ratings and a physiological stress marker.

Rehabilitation for patients with gastrointestinal cancer does not typically involve treatment for neurological or musculoskeletal deficits; rather, it focuses on preventing disuse syndrome induced by cancer treatment. Thus, this clinical scenario is particularly well suited to psychological evaluation using facial emotion analysis, subjective QOL assessment, and stress biomarker measurement. The observed psychological improvements—specifically, the increase in the Happy score and the decrease in the VAS stress score—underscore the value of incorporating psychological endpoints into routine cancer rehabilitation practice. Previous reports have demonstrated that supervised exercise therapy improves both psychological and physical function in cancer patients [33]. In our study, physiotherapist-supervised rehabilitation treatment tailored to each patient’s postoperative recovery also yielded psychological benefits, which aligns with previous findings. In addition, preoperative depressive status has been significantly linked to higher rates of postoperative complications [34]. Our individualized rehabilitation program guided by postoperative psychological assessment may have contributed to better postoperative recovery via the optimization of patients’ psychological state during hospitalization. Given that the improvement in psychological indices did not directly influence physical function outcomes, such as the 6MWD test or FTSST performance at discharge, early postoperative psychological and physical improvements likely do not occur in parallel.

Facial emotion analysis may offer advantages over traditional subjective assessments. The lack of correlation between facial emotion analysis and the VAS score suggests that the two approaches capture different facets of emotional responses. Subjective assessments include simple tools, such as the VAS and the Numerical Rating Scale, as well as multi-item questionnaires, such as the Short-Form 36 [35] and the EuroQol EQ-5D [36]. These are frequently used to assess QOL in cancer patients [37–40]. However, it has been reported that subjective assessments are easily influenced by respondents’ understanding of the questions and educational background, which may encourage avoidance of negative responses [41]. In this study, because the subjective assessment was intended to measure daily psychological changes alongside facial emotion analysis, we used a VAS to minimize this problem. Although simple tools, such as the VAS, are favored in clinical practice, their results should be interpreted with caution because single-axis rating scales can capture only a limited spectrum of emotions and psychological states.

Our finding that an increase in the Sad emotion score was accompanied by a rise in sAA level indicates a physiological link between negative affect and the stress response. Previous studies have reported that momentary self‑reported negative emotions in real-time correlate with elevated salivary cortisol [42,43], which is consistent with our results. However, sAA levels can be affected by various factors, such as food intake [44]. In upper gastrointestinal cancer surgery, patients often undergo brief periods of fasting for anastomotic protection. However, because we only assessed sAA levels immediately before and after the rehabilitation sessions, we were unable to control for potential confounding factors, such as eating status or diurnal variation. The absence of correlations between sAA level and other emotion category scores suggests that facial emotion analysis can detect emotional nuances that are beyond the scope of physiological markers.

Subjective assessments and physiological stress markers—such as the VAS scores and sAA levels used in this study—are established and reliable evaluation methods, yet they are impractical for assessing the wide range of human emotions. Facial emotion analysis allows the evaluation of emotions that conventional indices cannot measure. Rehabilitation protocols developed using quantitative emotional data may better align with each patient’s disease status, physical characteristics, and preferences; moreover, their ability to capture emotional changes in real time over a certain period offers an advantage over traditional assessment methods, such as the VAS and sAA measures.

This study was limited by its small sample size. We could only adjust for age as a covariate in the multiple regression analysis examining how emotional changes affect physical function. Therefore, the generalizability of the results is limited. Future work with a larger number of participants should include analyses that incorporate other potential confounders, such as sex, tumor stage, and preoperative test values. In addition, because atypical facial expressions may be induced by having a third party record the rehabilitation sessions, a single physiotherapist performed both the filming and training to replicate routine clinical conditions. Although this ensured the consistency of the intervention, we were unable to assess interrater reliability, and we may have also introduced evaluator bias, which could have affected the generalizability of our findings. Future studies should involve multiple evaluators and establish a suitable recording method for conducting real-time facial emotion analysis during rehabilitation treatment. Additionally, previous studies have shown that the accuracy of facial emotion analysis can differ among various software, even when applied to the same dataset [45]. Our previously reported case study [46] is the only published work to date using the MAL Face Emotion software, and the reliability and validity of this software are on the path to becoming established.

## Conclusion

Facial emotion analysis immediately before and after rehabilitation sessions in patients with upper gastrointestinal cancer revealed that the sessions improved the Happy emotion component. Although exercise therapy for cancer patients is known to induce a positive psychological effect, objective indicators for such an effect remain scarce. This study is the first to apply facial emotion analysis to the field of cancer rehabilitation medicine to quantify emotion. Facial emotion analysis is still an emerging discipline in which the scientific underpinnings are largely theoretical, and each step from image parameterization to analysis takes many different forms [47–50]. Nevertheless, it is notable that emotion assessment, which has traditionally depended heavily on clinicians’ intuition, can now be performed objectively and quantitatively. We anticipate its future application across a range of diseases and stages.

## Supporting information

S1 Checklist STROBE statement. (DOCX)

S1 File Dataset. (XLSX)
